# EPAD1 Orthologs Play a Conserved Role in Pollen Exine Patterning

**DOI:** 10.3390/ijms25168914

**Published:** 2024-08-16

**Authors:** Huanjun Li, Miaoyuan Hua, Naveed Tariq, Xian Li, Yushi Zhang, Dabing Zhang, Wanqi Liang

**Affiliations:** 1 Joint International Research Laboratory of Metabolic & Developmental Sciences, State Key Laboratory of Hybrid Rice, School of Life Sciences and Biotechnology, Shanghai Jiao Tong University, Shanghai 200240, China; 18213460953@sjtu.edu.cn (H.L.); hmy0565@sjtu.edu.cn (M.H.); naveedtariq@sjtu.edu.cn (N.T.); li_xian@sjtu.edu.cn (X.L.); jaccountaria@sjtu.edu.cn (Y.Z.); zhangdb@sjtu.edu.cn (D.Z.); 2 School of Agriculture and Biology, Shanghai Jiao Tong University, Shanghai 200240, China

**Keywords:** Poaceae, primexine, pollen exine pattern, *EPAD1*, apical spikelets abortion

## Abstract

The pollen wall protects pollen during dispersal and is critical for pollination recognition. In the Poaceae family, the pollen exine stereostructure exhibits a high degree of conservation with similar patterns across species. However, there remains controversy regarding the conservation of key factors involved in its formation among various Poaceae species. *EPAD1*, as a gene specific to the Poaceae family, and its orthologous genes play a conserved role in pollen wall formation in wheat and rice. However, they do not appear to have significant functions in maize. To further confirm the conserved function of *EPAD1* in Poaceae, we performed an analysis on four *EPAD1* orthologs from two distinct sub-clades within the Poaceae family. The two functional redundant barley *EPAD1* genes (*HvEPAD1* and *HvEPAD2*) from the BOP clade, along with the single copy of sorghum (*SbEPAD1*) and millet (*SiEPAD1*) from the PACMAD clade were examined. The CRISPR-Cas9-generated mutants all exhibited defects in pollen wall formation, consistent with previous findings on *EPAD1* in rice and wheat. Interestingly, in barley, *hvepad2* single mutant also showed apical spikelets abortion, aligning with a decreased expression level of *HvEPAD1* and *HvEPAD2* from the apical to the bottom of the spike. Our finding provides evidence that *EPAD1* orthologs contribute to Poaceae specific pollen exine pattern formation via maintaining primexine integrity despite potential variations in copy numbers across different species.

## 1. Introduction

Spores or pollen act as the reproductive propagules containing the parental genetic material in all terrestrial plants, which is enclosed within a thick spore/pollen wall providing protection against desiccating and other physical damage during dispersal. Spores are produced by non-seed plants, and their derived homologues pollen are produced by seed plants. Upon release from the anther, pollen undergoes dispersion by wind or other vectors such as insects before ultimately being deposited. During its journey, it is exposed to ultraviolet (UV) radiation, dehydration, and pathogens directly [1]. The pollen wall is a multi-layer structure that displays morphological diversity between species, which is associated with pollination recognition and also constitutes an important feature of plant taxonomic classification [2]. However, the pollen wall structure and development also share common features across plant taxa [3]. The pollen surface patterns are formed by the accumulation of sporopollenin, one of the most resistant organic macromolecules, which represents the key innovations for land plants in their colonization of terrestrial habitats. It is believed pollen wall morphology/pattern diversification occurred during species evolution, while the mechanism underlying the diversity of this regular pattern is largely unknown.

Pollen wall development initiated at the tetrad stage, with a thin microfibrillar matrix, primexine, formed between the microspore plasma membrane and the surrounding callose wall. Subsequent sporopollenin precursors deposition and polymerization are guided by the primexine. The composition of primexine closely resembles that of the primary cell wall, encompassing cellulose, pectin, and xylan, in addition to arabinogalactan-proteins and lipoproteins [4,5,6]. The specific components and mechanisms through which primexine directs exine patterning are not clear. Previous studies have demonstrated that the genes responsible for regulating sporopollenin precursors biosynthesis and primexine formation are highly conserved across land plants [7,8,9]. Although numerous molecular factors necessary for primexine formation have been identified, the biological functions of most of them remain unknown. These genes are conserved in flowering plants, while the factors that regulate species-specific wall patterning remain unidentified.

Poaceae, also known as Gramineae, is a highly diverse plant family of monocotyledonous flowering plants, commonly referred to as grasses. Interestingly, the Poaceae family shows a consistent stenopalynous pollen morphology, that is a spherical shape with a psilate surface and a monoporate aperture. While differences exist in pollen grain size, the size of the aperture and the specific surface ornamentation. Variations in pollen surface ornamentation mainly are the spinules size, density, and arrangement [10,11]. According to these characteristics, 15 pollen surface morphotypes, including *Anomochloa*-type, *Arthrostylidium*-type, *Avena*-type, *Diandrolyra*-type, *Hordeum*-type, *Muhlenbergia*-type, *Olyra bahiensis*-type, *Olyra juruana*-type, *Pariana*-type, *Poa*-type, *Setaria*-type, *Stipa*-type, *Streptochaeta*-type, *Sucrea*-type, and *Triticum*-type, are divided [10]. The diversity of pollen surface ornamentation is positive correlated with species richness at the subfamily level while most of the pollen surface ornamentation morphotypes of Poaceae (98.1%) remains unexplored, and the relationship between pollen surface diversification and species evolution is not clear.

Although the detailed pollen surface ornamentation is various, the three-dimensional structures are quite similar in Poaceae. The general process of pollen wall development is also highly conserved in plants [3]. Previously, we reported a Poaceae specific type-G lipid transfer protein, EPAD1, which plays an essential role in the patterning of rice pollen exine [9]. The mutation of EPAD1 orthologs in wheat, TaMS1 (*Triticum aestivum* L.) or ThMS1 (*Thinopyrum ponticum*), both result in male sterility [12,13,14]. Phylogenetic analysis has revealed that EPAD1 and orthologs are highly conserved in Poaceae. EPAD1 and orthologs specifically express in meiocytes and they all show phospholipids binding activity in vitro [9,12,13,15]. However, research in maize offers a distinct perspective, as two EPAD1 orthologs, ZmLTPg11 and ZmLTPx2, have been identified. Notably, even in the double mutant *zmltpg11*/*zmltpx2*, viable pollens are still produced. [15]. Thus, it remains controversial whether the EPAD1 orthologs function are conserved in Poaceae pollen exine patterning or contribute to the different pollen surface ornamentation among Poaceae.

The subfamilies of Poaceae are traditionally classified into three early diverging monophyletic groups: Anomochlooideae, Pharoideae, and Puelioideae, and two major clades: Bambusoideae, Oryzoideae, and Pooideae form the BOP clade, and Panicoideae, Arundinoideae, Chloridoideae, Micrairoideae, Aristidoideae, and Danthonioideae form the PACMAD clade [16,17]. BOP and PACMAD clades diverged from each other at 81.43 Ma and showed an increase in species richness during the Miocene period (<23 Ma) [18,19]. Rice and wheat are members of the BOP clade, while maize is classified into the PACMAD clade. Therefore, there is ongoing controversy regarding the function of EPAD1 and its orthologs in pollen exine patterning, as they appear to have a conserved role only in the BOP clade rather than in the PACMAD clade. In this study, a series mutant of EPAD1 orthologs were generated in Poaceae crops from two distinct clades, including barley from the BOP clade, and millet and sorghum from the PACMAD clade to address this question. Our work provides evidence that EPAD1 orthologs contribute to Poaceae-specific pollen exine patterning despite variations in the copy number in barley.

## 2. Results

### 2.1. Poaceae Pollen Morphology Is Conserved but Surface Ornamentation Is Diverse

Poaceae are extremely economically important as they are a primary source of staple foods derived from cultivated cereal crops such as wheat, rice, maize, barley, and millet. Additionally, they serve as critical feed for meat-producing animals. The classification and evolutionary history of the Poaceae family are obscured for their remarkable diversity. Based on the Grass Phylogeny Working Group II (GPWG II) system, two major clades, the PACMAD clade and the BOP clade, as well as three early diverging monophyletic groups are included in Poaceae. We searched the available pollen wall data from PalDat, and integrated into the Poaceae phylogenetic tree. It shows pollen exine patterns are generally similar across Poaceae species (Supplementary Figure S1).

*EPAD1* as a specific gene of Poaceae family has been reported to play an essential role in rice pollen exine patterning. To further elucidate the function of *EPAD1* orthologs in other Poaceae crops, we selected barley from the BOP subfamily, and millet and sorghum from the PACMAD subfamily for our study. EPAD1 protein sequence was used against each database to identify candidate genes; the results suggest there are two *EPAD1* orthologs in barley, termed as *HvEPAD1* (HORVU.MOREX.r2.4HG0277950) and *HvEPAD2* (HORVU.MOREX.r2.4HG0277970), as well as an ortholog from millet, termed as *SiEPAD1* (XP 004978021), and an ortholog from sorghum, termed as *SbEPAD1* (Sobic.006G089700) (Supplementary Figure S2).

### 2.2. Mutations of BOP Clade HvEPAD1 and HvEPAD2 Affect Pollen Development

The single mutants, *hvepad1* and *hvepad2*, and the *hvepad1*/*hvepad2* double mutant were generated by the CRISPR-Cas9 gene editing system. We obtained three independent alleles of *hvepad1* (*hvepad1-1*; *hvepad1-2*, and *hvepad1-3*), and two independent alleles for *hvepad2* (*hvepad2-1* and *hvepad2-2*). For the *hvepad1*/*hvepad2* double mutant, which was generated by the combination of *hvepad1-1* and *hvepad2-1*, see Supplementary Figure S3.

**Figure 1:**
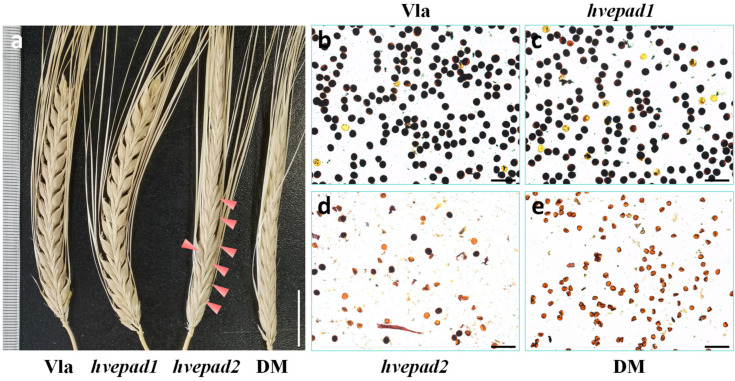
HvEPAD1 and HvEPAD2 function in male fertility. (**a**) Spikes of wide type Vlamingh (Vla), *hvepad1*, *hvepad2* and *hvepad1*/*hvepad2* double mutant (DM) at mature stage. Red arrowheads indicate viable barley seeds. Bar = 2 cm. (**b**–**e**) Staining with Lugol’s iodine solution of wild-type Vla (**b**), *hvepad1* (**c**), *hvepad2* (**d**) and *hvepad1*/*hvepad2* (**e**) mature pollen grains. Viable pollens are stained dark color. Bars = 100 µm.

*hvepad1* mutant developed a normal spike with a full seed set at the mature stage; the *hvepad2* mutant is partially fertile with few seeds on the mature stage spike, while the *hvepad1*/*hvepad2* double mutant is totally sterile (Figure 1a). Consistently, *hvepad1* could produce viable pollens, and fewer viable pollens produced by *hvepad2*, while *hvepad1*/*hvepad2* is totally male sterile (Figure 1b–e). These results suggest the *HvEPAD1* and *HvEPAD2* functions are redundant in barley, and mutations of them lead to male sterility.

### 2.3. Pollen Exine Patterns Are Defective in BOP Clade Orthologs Mutants

**Figure 2:**
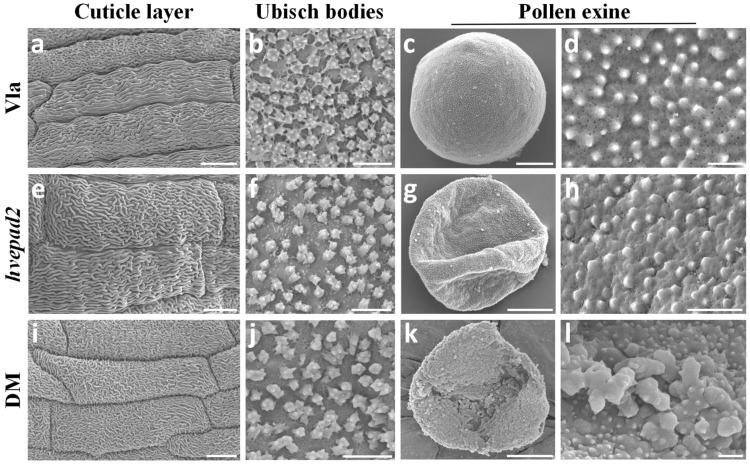
Pollen exine pattern is altered in *hvepad2* and *hvepad1*/*hvepad2*. (**a**,**e**,**i**) Scanning electron microscopy (SEM) images of the cuticle layer on the wild-type Vla (**a**), *hvepad2* (**e**) and *hvepad1*/*hvepad2* (**i**) anther surface. Bars = 10 µm. (**b**,**f**,**j**) SEM observation of Ubisch bodies on the inner surface of the wild-type Vla (**b**), *hvepad2* (**f**) and *hvepad1*/*hvepad2* (**j**) anther wall. Bars = 2 µm. (**c**,**g**,**k**) and (**d**,**h**,**l**) Pollen grains of wild-type Vla (**c**), *hvepad2* (**g**) and *hvepad1*/*hvepad2* (**k**) and the enlarged view of the wild-type Vla (**d**), *hvepad2* (**h**) and *hvepad1*/*hvepad2* (**l**) pollen surface. Bars = 10 µm in (**c**,**g**,**k**), and 1 µm in (**d**,**h**,**l**).

The rice *epad1* mutant and wheat *ms1* mutant showed impacts on the pollen exine pattern [9,12]. Scanning electron microscopy (SEM) was utilized to examine the pollen exine pattern in *hvepad2* and *hvepad1*/*hvepad2*. At stage 13, spherical pollen grains displayed an elaborate surface pattern with clear visible microchannels in barley wild-type plants showing the representative *Hordeum*-type ornamentation (Figure 2c–d). In *hvepad2*, the pollen surface exhibits irregular spinules and a rough texture, with microchannels embedded within the collapsed pollen exine (Figure 2g,h). In *hvepad1*/*hvepad2*, pollen exine defects were notably more severe, characterized by the presence of substantial sporopollenin-like aggregates on the surface, similar to those observed in the rice *epad1* mutant (Figure 2k,l).

We also examined the phenotype of anther cuticle layer and Ubisch bodies in *hvepad2* and *hvepad1*/*hvepad2*, all of which showed the normal lipidic structures. The cuticle layer displayed a spaghetti-like structure, while the Ubisch body showed a spur-like structure in barley (Figure 2a,b,e,f,i,j). These findings suggest that HvEPAD1 and HvEPAD2 have a specific impact on the patterning of pollen exine.

### 2.4. The Primexine Integrity Was Disrupted in BOP Clade Mutants

**Figure 3:**
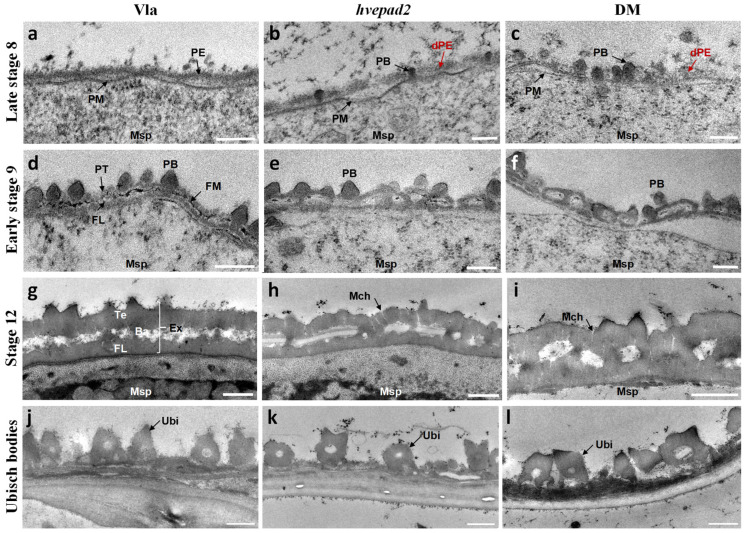
Integrity of the primexine layer is disrupted in *hvepad2* and double mutant, but Ubisch body formation is normal. (**a**–**i**) Transmission electron microscopy (TEM) observation of pollen exine of wild-type (**a**,**d**,**g**), *hvepad2* (**b**,**e**,**h**) and *hvepad1*/*hvepad2* (**c**,**f**,**i**) microspores at different developmental stages. (**j**–**l**) TEM observation of Ubisch bodies on the inner surface of the wild-type Vla (**j**), *hvepad2* (**k**) and *hvepad1*/*hvepad2* (**l**) anther wall. Vla, wide type Vlamingh; DM, *hvepad1*/*hvepad2* double mutant; Msp, microspore; PM, plasma membrane; PE, primexine; dPE, defective primexine; PB, probaculae; PT, protectum; FL, foot layer; FM, fibrillar material; Mch, microchannel; Te, tectum; Ba, baculae; Ex, exine; Ubi, Ubisch body. At least six biological replicates for wild type, *hvepad2* and *hvepad1*/*hvepad2* were used for TEM analysis of each stage (stages 8–12). Representative images are shown. Bars = 0.2 µm in (**a**–**f**), and 0.5 µm in (**g**–**l**).

To further investigate pollen exine defects more closely, we performed transmission electron microscopy (TEM) to observe the developmental process of pollen exine. In wild-type microspores, a continuous primexine matrix layer was deposited between the callose wall and the microspore plasma membrane at the tetrad stage (Figure 3a). At stage 9, the callose wall was degraded completely by tapetum-secreted callase, leading to the release of microspores into the anther locule. The pollen exine pattern showed a well-defined triple-layered structure, consisting of the protectum, fibrillar material, and the foot layer, with the probaculae arranged perpendicular to them in the wild-type (Figure 3d). This triple-layered structure guides the assembly of sporopollenin and leads to the development of a well-organized two-layered pollen exine, consisting of tectum, foot layer, and the baculae, at stage 12 (Figure 3g) while in *hvepad2*, the primexine exhibited compromised integrity with several fractures, and a loosened structure (Figure 3b). At stage 9, the triple-layered structure pattern was not able to be formed and the protectum and foot layer tended to fuse at certain points (Figure 3e). With the accumulation of sporopollenin, the fused tectum and foot layer are ultimately formed as the pollen exine pattern stage (Figure 3h). In *hvepad1*/*hvepad2*, the defects were more severe, with the reduction in primexine formation and fragmented, and probaculae randomly deposited on the primexine (Figure 3c). The typical triple-layered structure pattern was not observed, being replaced by numerous ellipsoidal structures (Figure 3f). At stage 12, thickened elliptical clumps appeared on the microspore surface (Figure 3i). Although the pollen exine pattern is defective in *hvepad2* and *hvepad1*/*hvepad2*, microchannels can be formed in both mutants (Figure 3h,i). These results suggest that instead of influencing the sporopollenin precursors assembly, HvEPAD1 and HvEPAD2 specifically affect the pollen exine pattern.

The Ubisch body and pollen exine develop synchrogenesis, and their surface ornamentation is often strikingly similar within a species [20]. Thus, we observed the Ubisch body structure using TEM at the pollen mature stage. Consistently, Ubisch bodies from wide type, *hvepad2* and *hvepad1*/*hvepad2* mutants showed a similar morphology (Figure 3j–l), indicating that HvEPAD1 and HvEPAD2 play a specific role in disrupting primexine integrity and thereby affecting pollen exine patterning.

### 2.5. HvEPAD2 Exerts an Impact on the Male Fertility of Apical Spikelets

**Figure 4:**
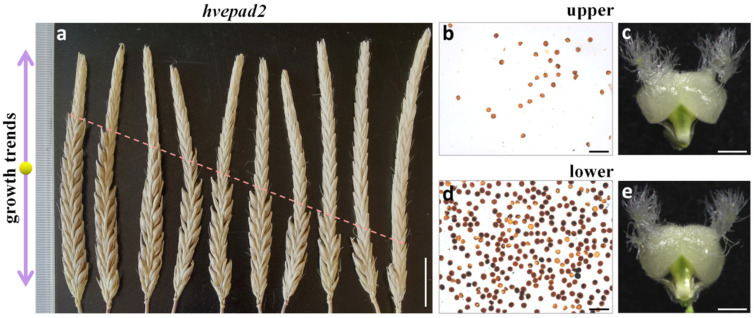
*hvepad2* panicles show apical spikelets infertility and less virable pollens in *hvepad2* apical spikelets. (**a**) Spikes of *hvepad2* at mature stage. Bar = 2cm. Double-sided arrow indicates the growth trends of barley spike. (**b**,**d**) Staining with Lugol’s iodine solution of *hvepad2* upper part (**b**) and lower part of the spike (**d**) mature pollen grains. Viable pollens are stained dark color. Bars = 100 µm. (**c**,**e**) Pistil of *hvepad2* upper part (**c**) and lower part of the spike (**e**) at mature stage. Bars = 1mm.

We noticed that the spikes showed infertility in the apical spikelets of *hvepad2*, despite the variability in seed setting rates (Figure 4a). Pollen production from the apical spikelets was dramatically lower than that from the bottom spikelets, while pistils were normal from both parts of the spikelets (Figure 4b–e), suggesting that male part sterility was responsible for the infertility of the apical spikelets. This result is consistent with the observed trend of the decreasing expression level of *HvEPAD1* and *HvEPAD2* from the apical to bottom spikelets, in which there was a relative higher expression of *HvEPAD2* compared to *HvEPAD1* (Supplementary Figure S4). We also attempted to validate the conserved function of barley EPAD1 orthologs through a genetic complementation assay, wherein we transformed the *HvEPAD2* genomic DNA sequence drived by rice *EPAD1* promoter into rice *epad1* mutant background. The results demonstrated that this construct was able to partially restore the male sterile phenotype in rice *epad1* mutants (Supplementary Figure S5). Our findings suggest that *HvEPAD1* and *HvEPAD2* show functional redundancy in the regulation of pollen exine patterning, particularly in the pollen development from the apical spikelets. Notably, *HvEPAD2* assumes a primary role attributed to its relative higher expression level.

### 2.6. Mutants of PACMAD Clade Genes Showed Similar Pollen Exine Defects as BOP Clade Mutants

**Figure 5:**
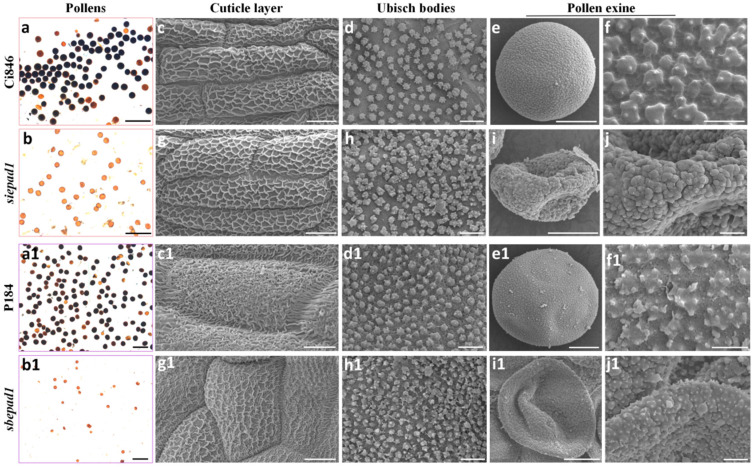
SiEPAD1 and SbEPAD2 function in male fertility. (**a**,**b**) Staining with Lugol’s iodine solution of wild-type millet Ci846 (**a**) and *siepad1* (**b**) mature pollen grains. Viable pollens are stained dark color. Bars = 100 µm. (**c**,**g**) SEM images of the cuticle layer on the wild-type Ci846 (**c**) and *siepad1* (**g**) anther surface. Bars = 10 µm. (**d**,**h**) SEM observation of Ubisch bodies on the inner surface of the wild-type Ci846 (**d**) and *siepad1* (**h**) anther wall. Bars = 2 µm. (**e**,**i**) and (**f**,**j**) Pollen grains of wild-type Ci846 (**e**) and *siepad1* (**i**) and the enlarged view of the wild-type Ci846 (**f**) and *siepad1* (**j**) pollen surface. Bars = 10 µm in (**e**,**i**), 1 µm in (**f**) and 2 µm in (**j**). (**a1**,**b1**) Staining with Lugol’s iodine solution of wild-type sorghum P184 (**a1**) and *sbepad1* (**b1**) mature pollen grains. Viable pollens are stained dark color. Bars = 100 µm. (**c1**,**g1**) SEM images of the cuticle layer on the wild-type P184 (**c1**) and *sbepad1* (**g1**) anther surface. Bars = 10 µm. (**d1**,**h1**) SEM observation of Ubisch bodies on the inner surface of the wild-type P184 (**d1**) and *sbepad1* (**h1**) anther wall. Bars = 2 µm. (**e1**,**i1**) and (**f1**,**j1**) Pollen grains of wild-type P184 (**e1**) and *sbepad1* (**i1**) and the enlarged view of the wild-type P184 (**f1**) and *sbepad1* (**j1**) pollen surface. Bars = 10 µm in (**e1**,**i1**), 1 µm in (**f1**) and 2 µm in (**j1**).

We also selected EPAD1 orthologs from the PACMAD clade species, *siepad1* (millet) and *sbepad1* (sorghum). Mutants were generated using the CRISPR-Cas9 system. Two alleles of *siepad1* mutant (*siepad1-1* and *siepad1-2*) and a *sbepad1* mutant were obtained (Supplementary Figure S3). Both *siepad1* and *sbepad1* mutants showed a male sterile phenotype (Figure 5a,b,(a1,b1)), which suggests SiEPAD1 and SbEPAD1 also play a role in pollen development, consistent with other EPAD1 orthologs in Poaceae.

SEM was then conducted to observe the mature stage anther and pollen surface in millet Ci846, *siepad1*, sorghum P184, and *sbepad1*. The cuticle layer of millet resembled a structure akin to a fishing net, and the spinules on the Ubisch body were relatively diminutive in size. Both the cuticle and Ubisch body of *siepad1* looked normal (Figure 5c,d,g,h). The sorghum cuticle layer showed a structure like spaghetti, similar to barley, although the not fully mature cuticle developed in *sbepad1* (Figure 5(c1,g1)). No obvious defect was observed of the spur-like Ubisch body in *sbepad1*, compared with sorghum P184 (Figure 5(d1,h1)).

The pollen surface ornamentation of millet displayed a *Setaria*-type, with few spinules arranged as groups (Figure 5e,f) while in *siepad1*, collapsed pollen was covered by sporopollenin globules, with no discernible microchannels (Figure 5i,j). Sorghum pollen surface sculpture elements assembled in a *Poa*-type (Figure 5(e1,f1)) while in *sbepad1*, the pollen surface showed irregular cracks without the presence of microchannels (Figure 5(i1,j1)). Together, these findings suggest that SiEPAD1 and SbEPAD1 have a specific impact on the patterning of pollen exine, and EPAD1 orthologs from the PACMAD clade species also play a conserved role in pollen exine pattern formation.

## 3. Discussion

### 3.1. Poaceae Pollen Exine Stereostructure Is Conserved but Surface Ornamentation Is Diverse

The durable pollen wall is a critical adaption for plants as they establish themselves in terrestrial environments. Pollen exine typically exhibits exquisite stereostructure and surface ornamentation. The fundamental structure of pollen exine is notably consistent across taxa, comprising tectum, foot layer (nexine), baculae perpendicular to these two layers, and a pollen coat (tryphine) filling in the gaps [8]. From the surface scene, the shape and surface feature are highly diverse, facilitating fertilization recognition and successful pollination.

The exine patterns of pollen show geometric diversity in different species, yet remain controlled and replicable within each specie [21]. The pollen exine stereostructure, including all fundamental structures, demonstrates a highly conserved pattern within Poaceae. However, the surface ornamentation exhibits diverse characteristics across the phylogeny. The diversity of surface ornamentation is positively associated with the species richness at the subfamily level in Poaceae, while the initial appearance of these morphotypes in the Cretaceous period (when Poaceae first evolved) or late Miocene, requires further study.

### 3.2. EPAD1 and Orthologs Function Conserved in Pollen Exine Patterning

The exine patterns of pollen exhibit diversity across taxa, but the underlying developmental processes are remarkably similar [3]. However, the mechanism responsible for the formation of these different patterns formation remains unclear. It is proposed that the diversity in pollen exine patterns may be led by primexine phase separation coupled to microspore plasma membrane undulations [21]. In the case of Poaceae pollen exine, the surface sculptural elements are relatively small and they give the appearance of a featureless pollen wall; however, Poaceae pollen exine shows an exquisite internal structure which was not considered by the phase separation model [9].

The pollen exine patterns of barley, millet, and sorghum were classified into different types based on their surface ornamentations, while the mutations of *hvepad1* and *hvepad2* in barley, *siepad1* in millet and *sbepad1* in sorghum all resulted in similar defective pollen exine patterns. Rice, barley, and wheat are classified in the BOP clade, while millet and sorghum are grouped in the PACMAD clade in the Grass Phylogeny Working Group II (GPWG II) system. Based on previous studies and our own findings, this clearly shows that EPAD1 and its orthologs from both clades in Poaceae play a conserved role in the development of pollen exine [9,12,13]. Interestingly, the mutation of two EPAD1 orthologs in maize (ZmLTPg11 and ZmLTPx2) does not affect pollen exine development [15], possibly due to the presence of other redundant genes. In barley, we also showed that pollen exine formation remains unaffected with the *hvepad1* single mutant, while there was compromised pollen exine with the *hvepad2* mutation, and a more severe pollen exine defect in the *hvepad1*/*hvepad2* double mutant. The complexity of the maize genome, with over 80% repetitive sequences, poses a significant challenge for sequencing and assembly [22]. However, recent advancements in sequencing technology have make it possible to achieve a telomere-to-telomere maize genome. Additionally, there is potential for the identification of other EPAD1 orthologs in maize in future studies.

### 3.3. The Gradient of Gene Expression Contributes to the Abortion of Apical Florets

The inflorescence development of cereal crops is spatiotemporally regulated, resulting in positional disparities in spikelets development. Along with variations in the developmental stages, there are obvious distinctions in nutrition assimilation, hormone distribution, and gene expression among spikelets located at variant parts of the spike [23,24,25,26,27,28,29]. Pre-anthesis tip degeneration or panicle apical abortion are common characteristics in cereal crops. This is a quantitative trait that is regulated by multiple genes of minor effects, meanwhile showing a strong interaction with environmental conditions [23,26,29]. Previous studies have documented numerous defects associated with panicle apical abortion or the ear barren tip [23,24,25,26,27,28]. For example, in rice, mutants leading to unbalanced endoplasmic reticulum (ER) stress signaling and increased ROS accumulation resulted in panicle apical abortion [28]. The development of the sterile tip in the maize ear is influenced by environmental factors and minor effect genes; however, no specific gene has been identified [26]. In wheat, most basal floret primordia undergo degeneration, with sugar deprivation triggering ABA and JA accumulation in the basal spikelet [27]. The integration of spatiotemporal transcriptome atlas with metabolome data elucidates a similar mechanism governing apical spikelet degeneration in barley [23,24,25].

In *hvepad2*, the apical spikelet failed to set seeds due to pollen sterility. The decreased expression level of *HvEPAD1* and *HvEPAD2* from top to bottom along the spike suggests a certain gene expression level is also spatiotemporal regulated during the spike development. Recently, Jiang et al. reported that ALOG (*Arabidopsis thaliana LSH1* and *Oryza G1*) members exhibit unequal expression levels in the upper-mid part and basal part of the spike, thereby contributing to the maintenance of the inflorescence architecture in barley [30]. In rice, SPL6 (*SQUAMOSA PROMOTER-BINDING PROTEIN-LIKE 6*), a transcriptional repressor, is highly expressed in the apical region of inflorescence, particularly in anthers. The mutation of *spl6* results in cell death in anthers of apical spikelets, suggesting that the function of *SPL6* is dependent on its expression level [28]. TUT1 (*TUTOU1*) acts as a suppressor of the cAMP receptor and plays an essential role in anther development. In the rice *tut1* mutant, aberrant anther and pollen development are observed in apical parts of the panicle [31]. *DPS1* (*Degenerated Panicle and Partial Sterility 1*) encodes a cystathionine β-synthase domain containing protein, which plays a regulatory role in maintaining ROS homeostasis. The rice *dps1* mutant showed small whitish anthers and degenerated apical spikelets [32]. Studies on barley have shown that anther typically undergoes degeneration before other organs are affected [23]. Integrating prior research with our findings elucidated the involvement of a spatial gene expression network in anther development, specifically its role in panicle apical abortion.

*HvEPAD2* is the first reported gene, which was associated with apical spikelets sterility in barley. Wheat as an allohexaploid, harbors three orthologs *TaMS1*, *TaMS1a* and *TaMS1d*, with *TaMS1a* and *TaMS1d* being epigenetically silenced during the generation and selection [13]. Barley is widely cultivated worldwide, yet its domestication period is significantly shorter than that of rice and maize. Consequently, more than 75% genes in barley display variant copy numbers, which are associated with the presence of segmental duplication (SD)-rich regions [33]. Copy number variants (CNVs) play a critical role in influencing important agronomic traits in barley, as well as in other cultivated crops [34,35,36]. In summary, the differential expression levels of genes along the spike/panicle play a critical role in contributing to apical degeneration in cereal crops, particularly through the regulation of anther development. This finding provides valuable insights for manipulating inflorescence architecture and enhancing yield potential in cereal crops.

## 4. Material and Methods

### 4.1. Plant Materials and Growth Conditions

Two-rowed barley (*H. vulgare*) cv. Vlamingh was used to generate CRISPR-CAS9 knock-out *hvepad1*, *hvepad2*, and *hvepad1*/*hvepad2* mutants. All barley plants were grown under 16 h photoperiod at 18 °C/15 °C (day/night) in a growth chamber. Foxtail millet (*Setaria italica*) cv. Ci846 and sorghum (*Sorghum bicolor* L.) cv. P184 were used to generate CRISPR-CAS9 knock-out *siepad1* and *sbepad1* mutants, respectively. All millet and sorghum plants were grown under 16 h photoperiod at 25 °C/22 °C (day/night) in a growth chamber. CRISPR constructs and monocot Agrobacteria-mediated transformations were conducted according to Ma et al., 2015 [37] and Hua et al., 2023 [38], and performed by technicians from the Chinese Academy of Agricultural Sciences. Primers used for CRISPR constructs are listed in Supplementary Table S1.

### 4.2. Phylogenetic Tree Analysis

A full-length EPAD1 protein sequence was used as the query for its closest relatives in published databases including the National Center for Biotechnology Information, Phytozome, Gramene, and EnsemblPlants. Protein sequences were aligned using MEGA 7, with a gap extension penalty and gap opening penalty set to 0.01 and 10, respectively. The phylogenetic tree (Supplementary Figure S2) was constructed based on multiple alignments of complete protein sequences.

### 4.3. Online Pollen Palynological Data

Poaceae pollen palynological data were searched from the PalDat – Palynological Database (https://www.paldat.org/, accessed on 30 June 2022) (Supplementary Figure S1). The Poaceae cladogram was drawn according to Grass Phylogeny Working Group II [17] and Poaceae–Wikipedia (https://en.wikipedia.org/wiki/Poaceae, accessed on 16 May 2022).

### 4.4. Characterization of Mutant Phenotypes

For all the phenotyping analyses, the spikes and florets were sampled only from the main culm of the plants. Spikes were photographed using an E995 digital camera (Nikon, Tokyo, Japan). Florets and pistils were photographed with an M205A microscope (Leica, Wetzlar, Germany). Pollen viability was analyzed under an Eclipse 80i microscope (Nikon) after pollen grains were released and immersed into Lugol’s iodine solution (2% [*w*/*v*] potassium iodide and 0.2% [*w*/*v*] iodine in water). Anther and pollen staging of barley was according to Gómez et al., 2012. In a short, spike range of 2–3 cm, pollen mother cells undergo meiosis; 3–4 cm, microspores release; 4–5 cm, free microspores; 5–6 cm, microspores become vacuolated; LFE1 (last flag elongation), mitosis I; LFE3, binuclear pollen; and finally LFE4, trinuclear pollen [39]. In millet and sorghum, florets just before flowering were collected for SEM analysis. We performed SEM analysis with mature stage anthers and pollens according to Li et al., 2020 [9] and examined with a JSM-7800F scanning electron microscopy (JEOL, Tokyo, Japan). Briefly, fresh samples were fixed in an FAA solution (3.5% formalin, 5% acetic acid and 50% ethanol). After a series of ethanol dehydration, samples were dried, attached to the conductive tape and coated with gold using EM SCD050 sputtering device (Leica). Anthers from different developmental stages were collected and fixed with 2.5% (*v*/*v*) glutaraldehyde in 0.1M phosphate buffer, after being washed three times with 0.1M phosphate buffer, then fixed in 1% (*w*/*v*) osmium tetroxide in 0.1M phosphate buffer. After another three washes with 0.1M phosphate buffer, samples were stained with 1% (*w*/*v*) aqueous uranyl acetate, then dehydrated through an ethanol series and embedded with TAAB Low Viscosity Resin (TAAB, T049; medium hardness). TEM sections were stained with 2% uranyl acetate and 2.6% (*w*/*v*) lead citrate aqueous solution and examined using G2 spirit Biotwin TEM (Thermofisher, Waltham, MA, USA).

### 4.5. Transcriptome Data

Transcriptome data of *HvEPAD1* (HORVU.MOREX.r2.4HG0277950) and *HvEPAD2* (HORVU.MOREX.r2.4HG0277970) were sequenced in our previous study [39] and downloaded from Supplemental Information (https://doi.org/10.1016/j.celrep.2023.113441) [24].

## Data Availability

All data are presented in this manuscript and Supplementary Materials.

## Supplementary Materials

The supporting information can be downloaded at https://www.mdpi.com/article/10.3390/ijms25168914/s1.
