# Supplementary material for: EPAD1 Orthologs Play a Conserved Role in Pollen Exine Patterning
Source: Int J Mol Sci. 2024 Aug 16;25(16):8914. doi: 10.3390/ijms25168914 (PMC11354838; doi:10.3390/ijms25168914)
Supplement: Supplementary file 1 [file ijms-25-08914-s001.zip › ijms-3111681-supplementary figures.pdf]

Supplemental Materials

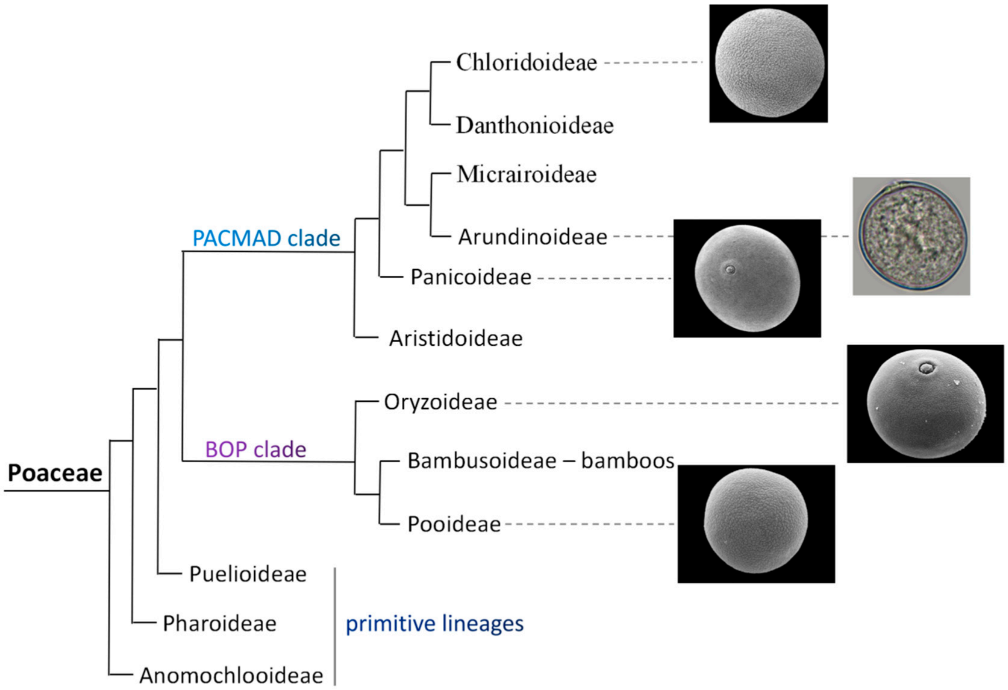

Supplemental Figure S1. A cladogram shows subfamilies and representative pollen morphology

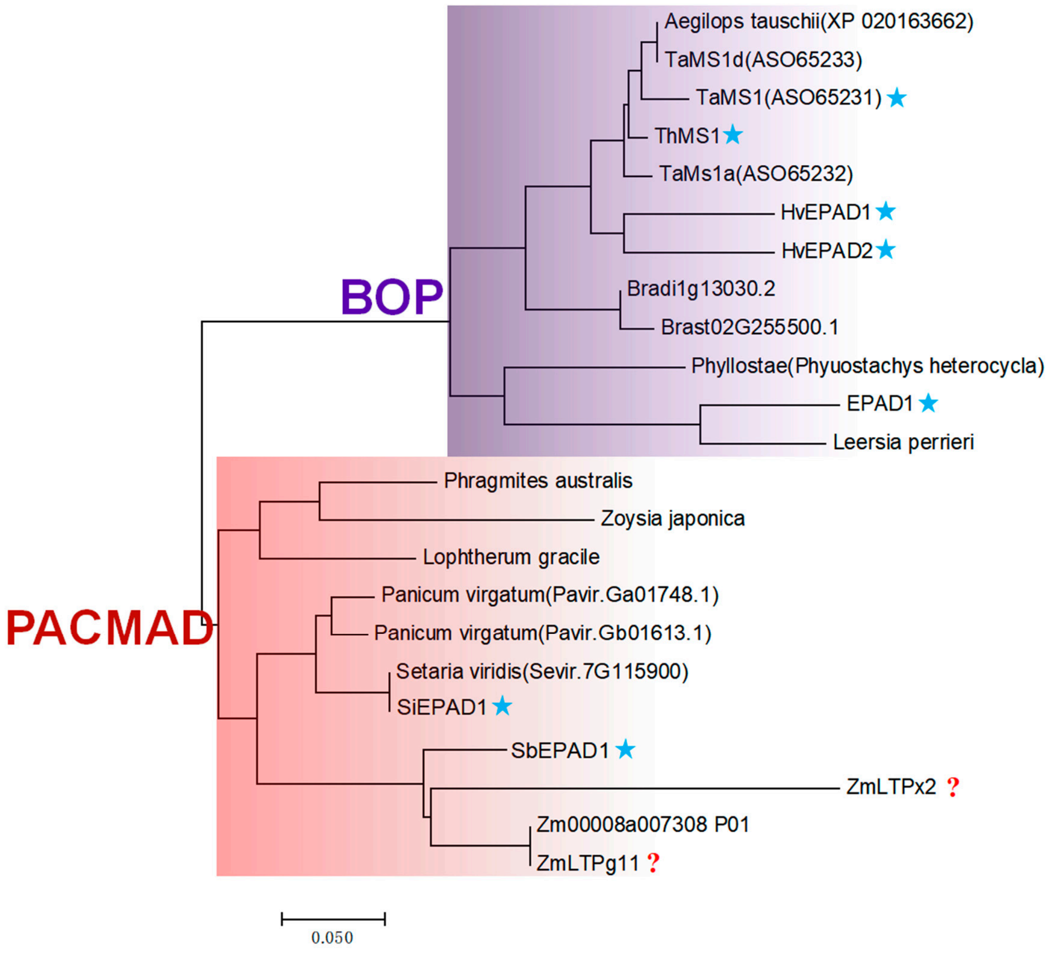

Supplemental Figure S2. Phylogenetic tree of EPAD1 and its orthologs from Poaceae

Stars indicate EPAD1 and its orthologs already studied include in this study. Scale bar indicates number of expected changes per amino acid residue.

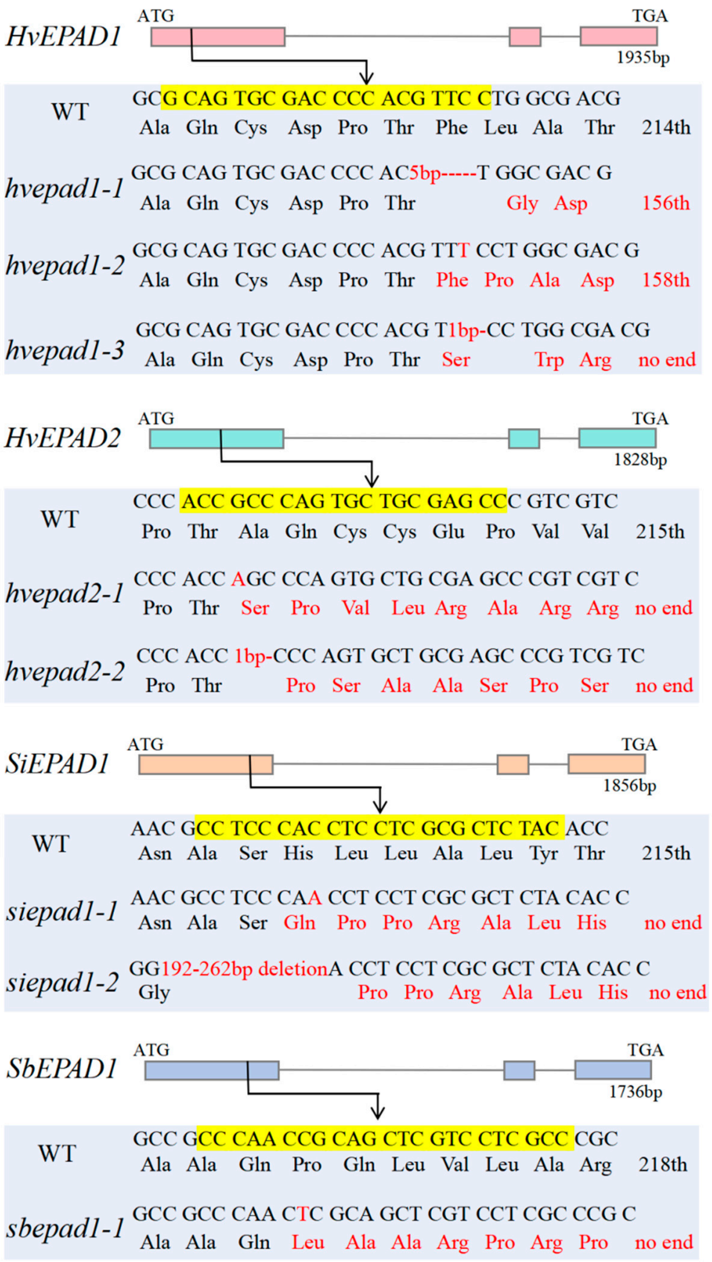

Supplemental Figure S3. Generation of *hvepad1*, *hvepad2*, *hvepad1/hvepad2*, *sbepad1* and *siepad1* mutants

CRIPSR/Cas9-mediated target mutagenesis of *HvEPAD1*, *HvEPAD2*, *SbEPAD1* and *SiEPAD1*. Three *hvepad1* alleles: *hvepad1-1*, 5bp deletion, resulting in premature termination; *hvepad1-2*, T insertion, resulting in premature termination; *hvepad1-3*, T deletion, resulting in frame shift. Two *hvepad2* alleles: *hvepad2-1*, G deletion, resulting in frame shift; *hvepad2-2*, T insertion, resulting in frame shift. Two *siepad1* alleles: *siepad1-1*, A insertion, resulting in frame shift; *siepad1-2*, 71bp deletion, resulting in frame shift. A *sbepad1* mutant: *sbepad1-1*, T insertion, resulting in frame shift.

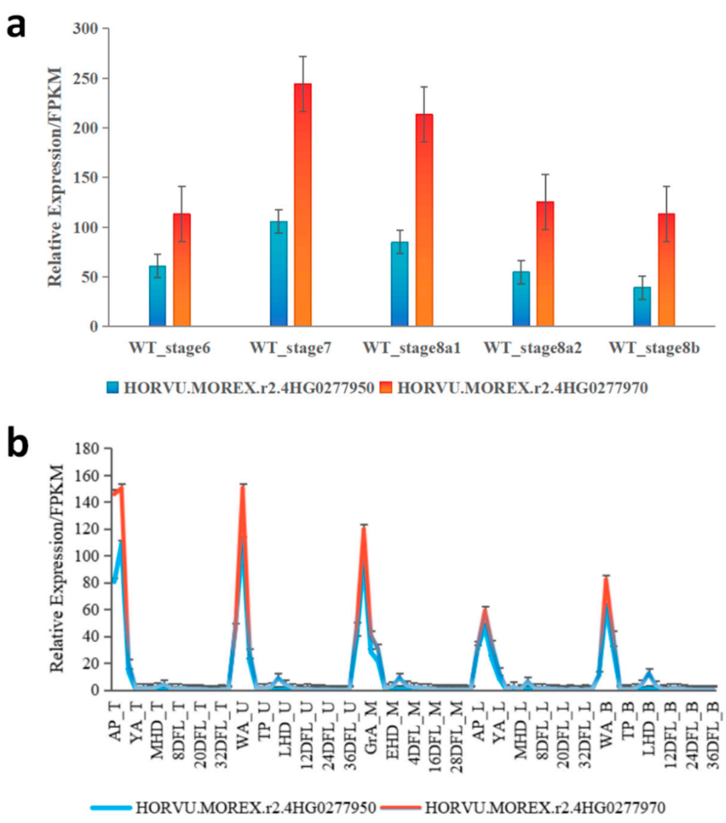

Supplemental Figure S4. Relative expression of *HvEPAD1* and *HvEPAD2*

(a) Relative expression of *HvEPAD1* and *HvEPAD2*. *HvEPAD1*, HORVU.MOREX.r2.4HG0277950; *HvEPAD2*, HORVU.MOREX.r2.4HG0277970. WT, wide type Vlamingh species. Error bars indicate SDs from three biological replicates.

(b) *HvEPAD1* and *HvEPAD2* express higher in top, upper middle and middle part then lower middle and bottom part of the spike. AP, awn primordium stage; WA, white anther stage; GrA, green anther stage; YA, yellow anther stage; TP, tipping stage; EHD, early heading stage; MHD, middle heading stage; LHD, late heading stage; DFL, day after late heading; T, the top of spike; U, upper middle (25% of spike length); M, middle (the center of spike length); L, lower middle (75% of spike length); B, the bottom of spike. Error bars indicate SDs from three biological replicates.

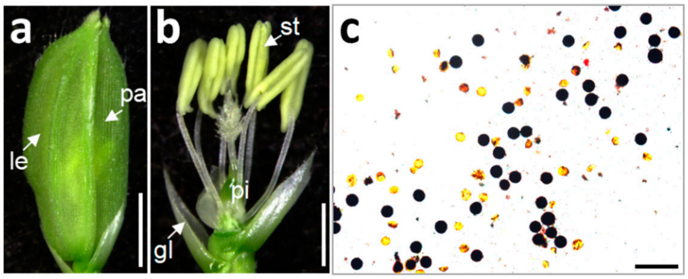

Supplemental Figure S5. ***HvEPAD2*** **can partially rescue** ***epad1*** **male fertility**

(a, b) Spikelet and after removal of the palea and lemma, showing normal floral organs. le, lemma; pa, palea; gl, glume; pi, pistil; st, stamen. Bar = 2mm in (a), and 1 mm in (b). (c) Staining with Lugol's iodine solution of mature pollen grains. Viable pollens are stained dark color. Bars = 100 μm.
